# Zebrafish as a Model for Fish Diseases in Aquaculture

**DOI:** 10.3390/pathogens9080609

**Published:** 2020-07-27

**Authors:** Louise von Gersdorff Jørgensen

**Affiliations:** Department of Veterinary and Animal Sciences, Faculty of Health and Medical Sciences, University of Copenhagen, DK-1870 Frederiksberg C., Denmark; lvgj@sund.ku.dk

**Keywords:** immunology, zebrafish, infection biology, prophylaxis, in vivo imaging, bacteria, viruses, parasites

## Abstract

The use of zebrafish as a model for human conditions is widely recognized. Within the last couple of decades, the zebrafish has furthermore increasingly been utilized as a model for diseases in aquacultured fish species. The unique tools available in zebrafish present advantages compared to other animal models and unprecedented in vivo imaging and the use of transgenic zebrafish lines have contributed with novel knowledge to this field. In this review, investigations conducted in zebrafish on economically important diseases in aquacultured fish species are included. Studies are summarized on bacterial, viral and parasitic diseases and described in relation to prophylactic approaches, immunology and infection biology. Considerable attention has been assigned to innate and adaptive immunological responses. Finally, advantages and drawbacks of using the zebrafish as a model for aquacultured fish species are discussed.

## 1. Introduction

### 1.1. Background

The zebrafish is extensively used as a vertebrate model for human diseases [1,2,3,4,5,6] and other conditions such as aging, development and genetics [7,8,9,10,11,12,13,14,15,16]. The small fish presents many advantages as a model; therefore, it cannot be overlooked and has not been since Dr. George Streisinger convinced the world in the 1980s of the wonders of the zebrafish [17,18,19]. The main advantages to date include optical transparency at early stages of life, a short generation time, continuous egg production, a large number of offspring, fast development, availability of a wide spectrum of mutant/transgenic lines, willingness of zebrafish scientists to share, ease of genetic manipulations and relatively cheap facilities. In particular, the ability to visualize processes inside the fish has consolidated this model as unique for intravital imaging—unmatched by any other animal model [13,20].

**Figure 1:**
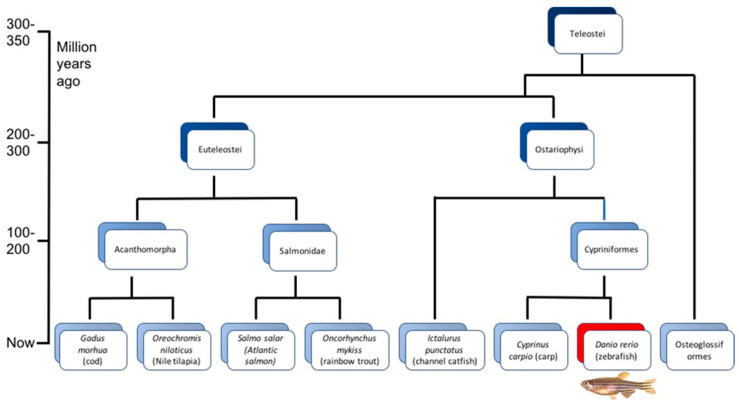
Evolutionary relationship between zebrafish and a selection of fish species used for production. Modified from Berthelot et al. (2014) [21].

Within the last two decades, the small fish has not only been used as a model for human conditions but also for fish conditions. In particular, fish diseases and immune responses have comprised the focus of the investigations when zebrafish were used as a model for fish. Relevant disease-causing pathogens are naturally those that are important for aquaculture productions and to utilize zebrafish as a model for fish appears straightforward. The genetic distance, however, is relatively large between some of the fish species and while zebrafish and carp belong to the same family, zebrafish and salmonids are quite distant (Figure 1). Nonetheless, zebrafish have contributed to important knowledge regarding fish diseases that are relevant for various production species and some of the investigations mentioned in this review have utilized the unique optical feasibility of real-time observation of processes in vivo. 

Fish disease outbreaks represent a key problem for the aquaculture industry as they can cause severe economic losses due to morbidity and mortality. A primary cause of such catastrophic epidemics is typically the high rearing densities used in modern intensive fish aquaculture, which facilitate the transfer and spread of pathogenic organisms. Chronic diseases are an additional concern in modern fish farming. Maintaining fundamental hygienic conditions as well as implementing vaccination strategies to improve fish health are, therefore, prerequisites for controlling diseases and should be included in management procedures. Regular surveillance of the health status of the farmed fish is also crucial for effective disease management, enabling a fast response to disease outbreaks and, as a consequence, reducing morbidity and mortality in the infected stocks [22,23]. New knowledge on immunological mechanisms and pathways as well as testing new treatments can, with relative ease and within a short period of time, be obtained in zebrafish and can benefit novel vaccine development and aid in treatment regimes.

The zebrafish belongs to the bony fishes (teleosts or Teleostei) together with most extant ray-finned fish. It belongs to the same family as carp (Cyprinidae) and is more distantly related to other fish used in production such as salmonids, channel catfish and cod (Figure 1). These fish and the zebrafish were separated evolutionary more than 150 M years ago [15], which is a relatively short time compared to the separation of mammals and teleosts, which took place approximately 400 M years ago. Zebrafish share the teleost genome duplication that occurred during the evolution of the ray-finned fish. They are, however, much smaller than fish used for production with a weight of 0.5–0.9 g and a fork length from 22–38 mm [23,24,25,26]. The females are able to spawn up to more than 200 eggs every 1–14 days [27,28], which is very useful for repetition of experiments and statistically powerful investigations. Zebrafish can furthermore survive at a temperature range of 6–38 °C but prefer ~28 °C [29].

The use of zebrafish as a model for aquacultured fish species has increased enormously since the review by Dham et al. 2006 [15] and Ribas et al. 2014 [23] and recent studies are included and described in this review. The majority of studies are conducted on bacterial and viral diseases (Table 1). Parasitic diseases are underrepresented (Table 1) but parasites are also more complicated to work with. Ectoparasites, for example, cannot be injected, which is common procedure for bacteria and viruses, while other parasites are species-specific and will not infect zebrafish. The lack of techniques to cultivate a wide spectrum of parasites in the laboratory represents another obstacle making experimentation problematic. Even infections with the most studied parasite in zebrafish, *Ichthyophthirius multifiliis*, which is of huge economic importance for the aquaculture industry, are more complicated to work with in the zebrafish model compared to classical infections in, e.g., rainbow trout (*Oncorhynchus mykiss*) [30,31,32], carp (*Cyprinus carpio*) [33,34] or channel catfish (*Ictalurus punctatus*) [35,36]. Zebrafish are more resilient and are only susceptible towards the parasite, when a stress factor is applied during the infection process. In the described studies, overcrowding was used and approximately 10 zebrafish per litre of water provided enough stress to facilitate ichthyophthiriosis. Zebrafish are naturally more resistant towards the parasite and this illustrates how even a fish to fish model can present species-specific differences related to infection biology and immune responses.

The research mentioned in this review on zebrafish as a model organism for aquacultured fish is focused on economically important diseases caused by bacteria, viruses and parasites and is divided into three focus areas; prophylactic approaches, immunology and infection biology. The studies were primarily focused on immunology represented with 42.5%, 61.8% and 57.1% of the papers on bacteria, viruses and parasites, respectively, whereas investigations on prophylactic approaches only represented 30%, 14.7% and 0%, respectively (Table 1). This distribution indicates that basic knowledge on immunological mechanisms in fish disease biology still is an ongoing and very important field for aquaculture research, while few prophylactic means are tested and perhaps species-specific characteristics prevent researchers from conducting vaccine experiments in the zebrafish model. Knowledge within fish immunology has lagged behind mammalian immunology because of missing tools. The opportunity for using front-line tools in zebrafish has probably contributed to the increase in papers on subjects such as infection kinetics and immunological mechanisms. There is an obvious desire for more knowledge and a deeper understanding of the induced immune responses by the pathogens with an aspect on how to use this information to combat the diseases in aquaculture.

### 1.2. Prophylactic Approaches

Treatments with chemicals, antibiotics or prophylactics have been the traditional way to fight fish infections. In recent times, however, vaccines for fish have emerged as the most efficient and promising solution [37,38]. Zebrafish must be susceptible towards the disease under investigation to represent a good model for prophylactic studies and several studies on protection have been conducted [39,40,41,42,43,44,45,46,47,48,49,50,51,52,53]. The zebrafish has also been used in a few studies for treatment purposes and has contributed with new knowledge for control of diseases [54,55].

### 1.3. Immunology

The immune system of fish is more primitive compared to humans [56]. Many basic functions and cell types are similar and fish also possess innate and adaptive immune mechanisms. Fish, however, do not have lymph nodes and the helper T-cell responses including Th1, Th2 and Th17 are not as clearly defined as they are in mammals [52,57]. Although it has been demonstrated that fish immunoglobulins are functional [32,58,59], the response of fish to immune challenges is strongly based on the innate immune response [56,60]. There are significant variations in the immune system between different fish species, and one of the more spectacular ones is that Atlantic cod (*Gadus morhua*) lacks the antigen-presenting major histocompatibility complex (MHC) II [61]. This molecule is, in other vertebrates, a part of the development of the classical adaptive immune response against bacterial and parasitic infections through the activation of CD4^+^ T-cells. MHC II malfunction is generally considered to lead to major immunodeficiency and death [62]. In 2005, a third group of antibodies, different to the classical fish antibodies IgD and IgM, called IgT/IgZ was identified in rainbow trout and zebrafish, respectively, and has since then been recognized in other fish species [63,64,65]. Rainbow trout also has multiple forms of C3 molecules as part of the complement system [66,67]. These immunological differences illustrate inter-species variation and emphasize that care has to be taken using one fish as a model for another fish species. For further information on the fish immune system please see [67,68,69].

During the first four days after fertilization, the zebrafish exhibits no adaptive immunity markers [70]. Many studies on innate immunology are conducted at this stage, both because no bias from adaptive immune mechanisms occurs and due to the fact that the larvae until 120 h post fertilization are not considered an experimental animal and no animal experimentation license is thus required. At four days after fertilization, expression of the genes activating recombination, *rag1* and *rag2*, ensues [71], and T and B cells are developed for future use in the adaptive system [72,73]. Complete functionality of the adaptive immune system takes 4 to 6 weeks to develop [74]. The zebrafish is highly useful as a model system in this regard because of the simplicity of using specific life stages to examine certain aspects of immunological maturation and function.

Bacterial and especially viral infections are associated with interferon (IFN) responses [48,75,76,77,78,79]. For example, an infection with infectious hematopoietic necrosis virus (IHNV) is often lethal and is linked to a delayed and inefficient IFN response [48]. Therefore, many studies focus on this molecule or other molecules influencing the production of IFN. The immunological investigations mentioned in this review have been dedicated to innate and adaptive immunity using classical transcription analyses [40,41,42,43,44,48,55,76,77,80,81,82,83,84,85,86,87,88,89,90,91,92,93,94,95,96], gnotobiotic (germ-free) [81] and transgenic fish lines for functional studies [89,91,97,98,99], but also visualization of the behaviour of professional phagocytes [100].

### 1.4. Infection Biology

Few investigations have been conducted on infection biology of important fish diseases in zebrafish. The model is, however, very suitable for this purpose, since it is feasible to visualize real-time in vivo how an infection spreads and how the immune system reacts. Transparent and gnotobiotic zebrafish have been used to study these aspects [82,101]. Different infection routes have furthermore been applied to study the natural and laboratory-induced kinetics of diseases [102,103,104]. Finally, pathogen tropism [105] and infections at different temperatures [106] have been investigated to learn the biology of the infectious agents.

## 2. Fish Diseases in Aquaculture Studied in the Zebrafish Model

### 2.1. Bacteria

Bacterial diseases in fish have caused problems as long as aquaculture has existed. Fish producers are currently facing the same problems as other animal producers with increasing resistance against antibiotics and other treatments [107]. Therefore, it is of vital importance to continue investigating the dynamics of bacterial diseases in fish, vaccines and the immunological responses of the fish to discover and develop new tools to control the infections. A summary of the studies mentioned in this section is found in Table 2.

#### 2.1.1. *Mycobacterium marinum*

*M. marinum* is a fish pathogen causing a chronic granulomatous disease similar to mammalian mycobacteriosis. The prevalence of *M. marinum* has increased worldwide with the intensification of fish farming and ornamental fish production [39] but the majority of studies using the zebrafish and *M. marinum* model have been conducted to study human medicine. A single prophylactic study documented that zebrafish immunized with a live attenuated L1D *M. marinum* mutant were protected following a challenge with a virulent *M. marinum* strain [39]. A recent study demonstrated that treatment with aspirin, which targets platelet activation, reduced the mycobacterial infection in zebrafish [54].

More research has been conducted on the immunological responses of zebrafish infected with the bacterium. Despite the fact that the embryos do not yet have lymphocytes, an *M. marinum* infection led to formation of macrophage aggregates with pathological signs of granulomas and activation of granuloma-specific *Mycobacterium* genes [108]. Therefore, infections in larvae initiated granuloma formation solely in the context of innate immunity [108]. A transcriptomic analysis showed that many genes related to immune responses, especially inflammatory genes, were up-regulated and the authors inferred that only some genes related to adaptive immunity were activated and that the reaction towards the bacterium therefore induced a specific response [80]. It has been shown that adaptive responses are critical to fight the pathogen. Rag1 mutant zebrafish, which lack the ability to activate recombination and thereby are deficient in functional B and T lymphocytes, were hypersusceptible towards the infection [97]. Reduction in the mycobacterial burden is dependent on macrophages and granuloma formation, which provides evidence that platelet activation induced by *M. marinum* compromises protective host immunity to the infection [54]. The usefulness of the transgenic line with fluorescently labelled macrophages and neutrophils [20] was recently demonstrated in a study visualizing in vivo dynamic processes in zebrafish larvae infected with *M. marinum*. Processes included immune cell migration, host/pathogen relationship and cell death [109]. Another transgenic line with interleukin-1β (Il1β) fluorescence was used to document early innate immune proinflammatory responses. In the same study, antimicrobial nitric oxide (NO) was shown to be involved in host protective mechanisms [98].

The zebrafish has also been used as a model for *M. marinum* pathogenesis and host/bacterium interactions [103,110,111]. Clinical pathology included the formation of granuloma-like lesions and the bacterium established either an acute or a chronic infection based upon inoculum. Infections were studied through the natural route using bath exposures and it was observed that the gastrointestinal track was the primary route of infection [102]. The *M. marinum* infection-induced lipid metabolism was furthermore studied using the zebrafish model [112,113].

#### 2.1.2. *Vibrio anguillarum*

*V. anguillarum* affects salt- and occasionally freshwater fish all over the world [114] and a relatively large amount of research has been conducted on this pathogen in zebrafish. Vaccination studies in zebrafish showed that the fish are able to acquire protection against *V. anguillarum* and a Th17-like response is induced following bath exposure with a live attenuated *V. anguillarum* strain [40,41,43]. It was documented that the live attenuated strain induced notable mucosal immune responses in the intestine with participation of neutrophils and macrophages [42]. The same research group tested a live attenuated combination vaccine against *Edwardsiella tarda* and *V. anguillarum* in zebrafish and turbot and achieved a high level of protection, especially against *V. anguillarum* [44]. Phage treatment also proved to be effective against vibriosis, when tested in zebrafish [115]. Some species of yeast isolated from the gut were used as probiotics against *V. anguillarum* infections and mortality was consequently reduced. It was suggested that gut colonization could be involved in the protective effect [116]. A functional compound, phenazine-1-carboxylic acid, derived from *Pseudomonas aeruginosa* strain PA31x was demonstrated to inhibit the growth of *V. anguillarum* and proved efficient as treatment against vibriosis in zebrafish [117].

Several studies using immunization with the live attenuated *V. anguillarum* have been conducted to investigate the immunological responses responsible for protection [40,41,42,43,44]. Following a challenge with wildtype pathogenic *V. anguillarum*, protection induced by the attenuated bacterium was confirmed and genes encoding pro-inflammatory factors such as Il1 and Il8 were found to be up-regulated 1–7 days post-vaccination, while the expression of *mhcII* increased 7 days post-vaccination [40]. The triggering of a MyD88-dependent signalling pathway in the intestine implied that the flagellum was the most important antigen in the attenuated vaccine. Professional phagocytes were found to participate in antigen recognition and sampling following vaccination and inflammation was observed in the intestine [42]. Furthermore, genes encoding factors participating in the Th17-like pathway were found to be up-regulated in the spleen and in mucosal tissues [41,43] and a Toll-like receptor and Mhc I and II signalling pathways were activated in the spleen and liver [44]. Another study using gnotobiotic zebrafish larvae revealed a downregulation of genes encoding Nfκb, Il1β, Mpo, Tlr4, Tlr22 and the authors suggested that *V. anguillarum* eluded the larvae’s innate immune defences as a “stealth mechanism” during the first stages of infection [81].

The infection kinetics of *V. anguillarum* in zebrafish have been investigated using transparent zebrafish to visualize the spread of a green fluorescent protein (GFP)-tagged *V. anguillarum* [101]. The bacterium was found to initially infect skin and intestinal surfaces. Another study used gnotobiotic zebrafish larvae and found colonization of the intestine [81]. Spread of infection with *V. anguillarum* was also compared through bath exposure and peritoneal injection [104]. The pathogen was found in the blood at all sampling points after injection, whereas only mucosal surfaces were affected initially following infection by bath. The authors emphasized, based on the results, how important mucosal immune defence mechanisms are. A similar study described that skin-injured zebrafish were more susceptible to a *V. anguillarum* infection and that the infection in such cases travelled much faster to internal organs and the blood stream [118].

#### 2.1.3. *Aeromonas salmonicida*

Infections with *A. salmonicida* in Norway comprised a major problem for the salmon industry until a vaccine was developed in 1993 [119]. The bacterium still causes problems all over the world and vaccines are not equally effective in all countries. A few studies solely on immunology have been conducted with this pathogen in zebrafish. One study described that infections with *A. salmonicida* induced expression of genes encoding the acute phase proteins serum amyloid a (Saa), Hepcidin and Haptoglobin [82], which is similar to the response of, e.g., salmon infected with the same pathogen [120]. This correlation indicates that zebrafish may be a suitable model for *A. salmonicida* infections. Intelectins also take part in the response against the bacterium in zebrafish [83] and following injection with a live *A. salmonicida*, significant mast cell degranulation was observed [121].

#### 2.1.4. *Yersinia ruckeri*

*Y. ruckeri* is a freshwater bacterium that infects salmonids [122]. Fish are routinely vaccinated against it but vaccines or management procedures are not optimal and there is room for improvement. A few studies have been conducted with *Y. ruckeri* in zebrafish. An inactivated vaccine was produced from a GFP-tagged *Y. ruckeri* and antigen uptake following bath immunization was visualized in transparent zebrafish at different life stages [45]. Common for all stages was that the intestine was a major location of antigen processing.

One study focused on the protective immunological response in zebrafish infected with *Y. ruckeri*, which was found to require Ifn-γ [84].

The infection biology of *Y. ruckeri* has also been investigated and the mode of action of the toxic effector antifeeding prophage 18 of the prophage tail-like protein translocation machinery was found to impair blastomere cell behaviour in zebrafish embryos [123].

#### 2.1.5. *Flavobacterium psychrophilum*

This bacterium is responsible for the bacterial cold-water disease and the rainbow trout fry syndrome in freshwater salmonids. Only one study was found on this pathogen in the zebrafish model and this may be due to the bacterium preferring temperatures below 16 °C [124]. Two bacterins based on different *F. psychrophilum* isolates were tested and differences in innate immune responses visualized by neutrophil migration in zebrafish larvae were found [46].

### 2.2. Virus

No naturally occurring virus has been discovered in zebrafish [125,126]; however, a range of fish viruses are nonetheless able to infect both larval and adult zebrafish. Similar to *F. psychrophilum*, the optimal temperature is often less than the 28 °C preferred by zebrafish. Despite that, much has been learned about the zebrafish viral immune responses, the infection biology and the pathologies of the viruses. In this review, four important viral fish diseases have been included (Table 3); spring viraemia carp virus (SVCV), IHNV, infectious pancreatic necrosis virus (IPNV) and viral haemorrhagic septicemia virus (VHSV). They are all RNA viruses and SVCV, IHNV and VHSV belong to the rhabdoviridae family, whereas IPNV belongs to the family birnaviridae.

#### 2.2.1. SVCV

SVCV is prevalent worldwide and is associated with haemorrhaging in cyprinids, especially in common carp (*Cyprinus carpio*) [127,128,129]. SVCV outbreaks usually occur during the spring, when the water temperature rises [130], and cause high mortality in young fish, with mortality rates up to 90% [127], leading to significant economic losses for the aquaculture industry. The vast amount of studies on SVCV utilizing zebrafish as a model appears reasonable, since carp and zebrafish belong to the same family and are thereby closely related (Figure 1). Basic biological features are thus very conserved between the two species. Only a few studies on preventive measures have been conducted and Encinas et al. (2013) identified, using a pathway-targeted microarray, genes and transcription factors implicated in viral shutoff and/or host survival responses after SVCV infection, which may contribute to the development of novel drug-based prevention methodologies [55]. A second investigation discovered that zebrafish beta-defensin 2 (zfBD2) has antiviral activity, immunomodulatory properties and is a potent viral DNA vaccine molecular adjuvant [47].

Several studies on evolutionary aspects of the immune system have been conducted in zebrafish challenged with virus and these include studies of type III Ifn as the ancestral antiviral system of vertebrates [75]. Functional studies have also been conducted for Ifns in zebrafish and two types of Ifns were shown to be induced after challenge with SVCV and IHNV and it was demonstrated that the different Ifns bound to two different receptors [76,77]. It has furthermore been shown that even though larvae possess protective antiviral Ifns, three-day old larvae were unable to mount a protective response following infection by the natural water-borne route [85]. Ifn-induced proteins with tetratricopeptide repeats (Ifits) also have conserved antiviral functions as in humans and were induced in zebrafish after challenge with SVCV [86]. In mammals, interferon regulatory factors (IRFs) regulate interferons. Zebrafish were utilized to demonstrate that zebrafish Irf4 was regulated by signal transducer and activator of transcription 6 (Stat6) and c-Rel [88] and that fish and mammals have evolved a similar Irf-dependent regulatory mechanism, fine-tuning Ifn gene activation [92]. An inhibitory effect was observed for some zebrafish Irfs at lower concentrations and a synergistic effect at higher concentrations [92]. An Ifn-inducing substance, 7-(6-(2-methyl-imidazole))-coumarin (D5) was found to elicit an innate immune response in non-viral infected zebrafish by up-regulating the expression of interferon genes (*Ifnγ*, *Ifnφ1*, *Ifnφ2* and *Rig-1*) and to inhibit SVCV replication after administration in infected fish [93]. Genes and transcription factors involved in different pathways have been identified, which were suggested to be implicated in suppression of the virus and/or host survival responses [55]. Zebrafish larvae were employed to visualize the damage caused by an infection with SVCV. Cellular processes, such as transendothelial migration of leukocytes, were demonstrated and virus-induced pyroptosis of macrophages and Il1β release could be observed in individual cells. In the zebrafish model, it was possible to identify exactly which cells were infected with the virus. Detailed host/pathogen interactions were discovered and the results of the study provided a more thorough understanding of the immune mechanisms implicated in the disease [99]. The negative regulators of LPS signalling, Md1 and Rp105 form complexes that directly interact with the Md2-Tlr4 pattern recognition receptor complex. A functional study using genetic inhibition of zebrafish Md1 and Rp105 revealed that Md1 or Rp105 deficiency impaired the expression of genes encoding pro-inflammatory and antiviral molecules. This led to increased susceptibility to viral infection and it was thereby demonstrated that these molecules had an important function for the regulation of innate immunity [87]. Nk-lysins are antimicrobial proteins produced by cytotoxic T lymphocytes and natural killer cells with a broad antimicrobial spectrum. Out of four identified *nk-lysin* genes in zebrafish, two were found to be up-regulated following an SVCV challenge [89]. Perforins are known in mammals to be involved in granule-dependent cell death. Genes encoding 6 perforins were identified in the zebrafish genome and one, Prf19b, which is mainly produced by myeloid cells, was involved in an antiviral defence, inducing protection after an in vivo infection with SVCV [90]. In some viral diseases, the viruses use Tnfα to their benefit. After a challenge with SVCV in zebrafish larvae, it was found that Tnfα blocked the host autophagic response, which is required for viral clearance [91]. A proteomic approach disclosed that proteins of the vitellogenin family (Vtg) and the grass carp reovirus-induced gene (Gig) proteins were up-regulated during SVCV infection, highlighting that these proteins are important in the antiviral response.

Only a few studies have been conducted in zebrafish on the infection biology of SVCV. It has been demonstrated that zebrafish were susceptible to infection by SVCV at 15–24 °C through the natural route—the water body—when 10(3) to 10(5) plaque-forming units per millilitre (PFU/mL) of water was used. Mortality was highest at the lower temperatures [106]. A transcriptomic analysis described changes and tissue-specific impacts caused by SVCV in vivo, which brought the understanding on host/pathogen interactions forward [131].

#### 2.2.2. IHNV

The first study in zebrafish on infections with IHNV was conducted in 2000 [132]. Adult zebrafish were infected with IHNV and IPNV and progression of the diseases was compared. It was found that the kinetics of hematopoietic defects between IHNV and IPNV infections diverged but common for both diseases were that the fish recovered 6 days after infection. IHNV is associated with a delayed and insufficient Ifn response and is normally lethal. A typical signature of Ifn-stimulated genes (Isgs) was observed in another study after challenge with IHNV and Chikungunya virus (CHIKV) in zebrafish larvae, but was stronger after challenge with CHIKV. Some inflammatory genes were induced through Ifn-independent pathways by IHNV and not by CHIKV and the fish recovered from CHIKV but not from IHNV. It was therefore demonstrated how host/virus interactions in zebrafish led to protective and non-protective antiviral innate immune responses [48].

Infection biology of this virus in zebrafish has been investigated to a limited extent. A novel form of zebrafish fibronectin (Fn2) on the cell surface was found to mediate IHNV attachment and cell entry [133]. Ludwig et al. (2011) fully explored the benefits of using zebrafish as a model to study IHNV infections. The authors took advantage of transgenic lines and visibility in the larval stage and described primary targets of the virus, reversibility of viral damage and spread of the disease in a whole vertebrate body [105].

#### 2.2.3. VHSV

VHSV is one of the most economically important viral diseases of rainbow trout and other farmed fish species [134]. In 2006, the first study of a VHSV infection in zebrafish was conducted and adult zebrafish proved to be susceptible via the injection and the bath route at 15 °C [49]. An attenuated virus was used for immunization and induced protection against VHSV at 15 °C, illustrating that zebrafish are able to mount a protective response even at a low temperature. A study on adjuvant efficacy has also been conducted using zebrafish and VHSV infections [50].

Five studies have focused on the immune response of zebrafish against VHSV. The first study in 2010 used gene expression analyses and described genes (such as those encoding complement components) that contributed to the early molecular events occurring in the fish surfaces during initial infections [94]. At the same time, it was discovered that temperature significantly affected the immune response of the fish. At 15 °C, zebrafish did not show altered gene expression after challenge, which they did at 28 °C. Furthermore, it was shown that Mx was important in the innate anti-viral response in the larvae [95] and that zebrafish IgM antibodies were key players in the acquired immunity [53]. A later study in 2015, also using expression data, uncovered that long-term VHSV survivors maintained molecular/cellular memories of viral encounters by modifying the expression levels of innate multigene families and, at the same time, had specific adaptive antibodies [96]. In our laboratory, we are currently investigating, using the transgenic line with fluorescently marked neutrophils, how these cells take part in the battle against VHSV and/or control the damage induced by the virus.

Investigations of VHSV infection biology have been conducted in several fish species [135] and this may be the reason for finding only a few studies in zebrafish. Regarding virulence, it was found that specific residues in the 3’-UTR of VHSV have a promoter function and are important for virulence in cells and pathogenicity in fish [136]. We are currently also investigating the kinetics and the tropism of the virus in vivo by inoculating zebrafish larvae with a transgenic VHSV virus, which induces red fluorescence in infected cells.

#### 2.2.4. IPN

In 1977, the first study using zebrafish as a model for viral infections was conducted. Results showed that injected IPNV was transmitted to the eggs (vertical transfer), and that this transmission occurred via females alone [137]. It was later confirmed that zebrafish also could acquire the infection by the natural route through the water body [132].

### 2.3. Parasites

Only a few investigations have been conducted in zebrafish with parasites, which are economically important for the fish production industry (Table 4).

#### 2.3.1. *I. multifiliis*

*I. multifiliis* is a single-celled freshwater parasite and a major problem all over the world in fish aquaculture [139]. The parasite is a generalist capable of infecting many species of fish and can, within two weeks, cause up to 100% mortality in production facilities. From 2016, several studies have described the immune response of zebrafish against *I. multifiliis* and it was documented that zebrafish were susceptible towards the parasite and were able to acquire immunological immunity [51]. Subsequently, regulation of immune-relevant genes was investigated and the main findings included an upregulation of the Th2-associated genes *il4/13* and *ighm* in immune fish and *il4/13* and *Saa* in naïve fish [52,140], which similarly have been observed in fish species used in production [31,32,141,142]. The transgenic line of zebrafish expressing GFP in connection with neutrophils has also been exploited to investigate the cellular responses against the parasite as well as host/parasite dynamics [100]. Neutrophils were highly attracted to infected sites 24 h after infection (quantified by counting an increase in the number of neutrophils) but the number of cells decreased during the next two days even though the parasites grew bigger and caused more damage. A video recording showed that the parasites ingested and neutralized functionally active neutrophils and thereby reduced the number of these cells. The most recent study investigated gene expression and the neutrophil response in naïve and *I. multifiliis*-immune zebrafish [52]. The number of neutrophils attracted to infection loci were highest in protected fish 24 h after infection even though it was documented that virtually no parasites were left on those fish, which was in contrast to naïve fish that hosted many parasites. It was hypothesized that parasites in the naïve fish ingested a large number of neutrophils, which resulted in the observed reduction in the number of cells. The gene *cxcl8*, which encodes a known chemoattractant of neutrophils, was not upregulated in the tissues with accumulations of neutrophils and a Cxcl8-independent sub-population of neutrophils was proposed to have comprised the responders.

The zebrafish was used as a host to study the infection biology of *I. multifiliis* for the first time in 2003. Here, zebrafish were found to be less susceptible to the parasite than channel catfish and it was concluded that zebrafish were suitable as a more resistant model for this disease [143].

#### 2.3.2. *Trypanosoma carassii*

Trypanosomes cause sleeping sickness in humans and a close relative *T. carassii* is a natural parasite of carp, zebrafish and some non-cyprinid freshwater fish. In 2019, visualization of the swimming behaviour of *T. carassii* parasites was documented in zebrafish for the first time [144].

## 3. Discussion

From the number of studies discussed in this review, the use of zebrafish as a model has contributed tremendously to knowledge on bacterial and viral diseases that are important for fish aquaculture. This is valuable for a more comprehensive understanding of host/pathogen interactions as well as for development of novel control measures. For both types of diseases, transcription analysis is a common tool to decipher immunological responses but with regard to bacterial diseases, the use of fluorescence-tagged bacteria is especially suitable to study the infection biology and real-time local immune responses in vivo in the zebrafish model. Only recently has such an approach been applied for viruses and such investigations may be more frequently encountered in the future. Studying host/parasite relationships in the zebrafish model has only recently begun. The applicability of the model may, however, never reach that of bacterial and viral diseases because of the difficulties working with parasites. Nonetheless, the model has already contributed with unique data, which are impossible to obtain in other fish species.

### 3.1. Advantages of Using the Zebrafish as a Model

The use of the robust zebrafish as a model represents obvious advantages compared to experimentation in the much larger fish species used for production. The small size benefits especially analyses of whole-body immune responses or infection patterns using technologies such as in vivo imaging (which can be conducted to cellular resolution), whole-mount in situ hybridization or whole-mount immunohistochemistry. The transparency of the larvae represents another advantage for visual documentation of, e.g., pathogen tropisms or tissue damage as a consequence of pathogen assault. To fully unravel the complicated host/pathogen interactions, real-time in vivo experimental infections are a prerequisite. Some studies use zebrafish as a model because the fish is small and easy to manage and breed and do not utilize any of the unique tools available. These tools (in vivo imaging, transgenic lines including fluorescence-labelling of cells, receptors, signalling molecules or tissues) are not widely available in other fish species. A variety of transgenic lines have been utilized in the mentioned studies but other relevant lines are available such as the one resistant to viral or bacterial infections [23,145,146], providing a tool to further study immune mechanisms in relation to disease. The more frequently used transgenic strains are those with a fluorescence marker on neutrophils and/or macrophages [20], where innate cell behaviours in vivo can be analysed, which is otherwise impossible in production fish. Another point of interest is that gnotobiotic zebrafish have been produced and exploited to follow microbial infections of sterile larval fish in real-time.

Zebrafish are able to survive at temperatures from 6–38 °C, making them perfect to study infections at various temperatures. Consequently, fish pathogens thriving at temperatures on both sides of the preferred 28 °C can be studied in the zebrafish and a few studies mentioned in this review took advantage of this.

Apart from transferring specific results of zebrafish genomics, biology and immunology to aquacultured species, a prospect of transferring research approaches such as morpholino-induced knockdown, the CRISPR knockout technology and transgenesis is also valuable.

The genetic similarity between zebrafish and production fish is greater than between zebrafish and mammals and the small fish is widely used as a model for mammalian biology and therefore, appears even more appropriate as a model for other fish species when taking species-specific differences into account. The zebrafish seems to be an ideal model for carp since the genetic distance between these species is so small.

### 3.2. Drawbacks of Using the Zebrafish as a Model

Even though the fish species to species genetic variation is less than from zebrafish to mammals, differences do exist and zebrafish are, for example, less disposed for stress and anatomically different from rainbow trout [147]. The zebrafish does not possess a true stomach [148], potentially reducing the applicability of results to aquacultured species that have one. However, enzymes specific to the vertebrate stomach are represented in zebrafish regardless of its anatomy [149]. A drawback in some cases is its small size, which makes it difficult to obtain blood in sufficient amounts and limits the amount of tissue and cell populations available for analyses. Some immune cell types such as natural killer cells are poorly characterized [78].

The feasibility of acclimatizing zebrafish to a desired temperature also comes at a price. Conducting investigations at a lower or higher temperature than 28 °C may have a consequence on the ability of the zebrafish immune system to react properly and a biased response may be expected, as well as a biased period of time until morbidity or mortality due to a pathogen is reached.

Breeding protocols are not standardized between facilities and inbreeding and outbreeding crosses may compromise the fitness of the zebrafish. As a consequence, experiments may be difficult to repeat from one laboratory to another [150].

The zebrafish may not be useful as a model for marine fish species such as Atlantic salmon (*Salmo salar*) under certain conditions. It does not tolerate salinities of more than approximately 10 ppt (personal observation) and the economically important salmon louse (*Lepeophtheirus salmonis*), which thrives at salinities >27 ppt [151], appears problematic to study in zebrafish.

## 4. Conclusions

There are more advantages than drawbacks using the zebrafish as a model for diseases in aquacultured fish species and much has been learned within the last couple of decades. Especially when the unique tools in zebrafish are exploited in vivo in real-time, novel host/pathogen interactions are documented and immune mechanisms are disclosed. Studying processes “live” in a whole vertebrate organism reveal novel insights contributing to basic science but hopefully, also contributing with valuable knowledge for the development of new treatments and prophylactic means.

## Figures and Tables

**Table 1:** Published papers mentioned in this review divided into three different focus areas.

Focus Area	Bacteria		Virus		Parasites	
	N°	%	N°	%	N°	%
Prophylactic approaches	12	30	5	14.7	0	0
Immunology	17	42.5	21	61.8	4	57.1
Infection biology	11	27.5	8	23.5	3	42.9
Total	40	100	34	100	7	100

**Table 2:** Studies on selected bacterial pathogens relevant for aquaculture utilizing zebrafish as an in vivo model.

Pathogen	Focus Area	Zebrafish Stage	Reference
*M. marinum*	Prophylactic approaches	Adult Larvae	Cui et al. 2010 [39] Hortle et al. 2019 [54]
	Immunology	Larvae Adult Adult Larvae Larvae Larvae	Davis et al. 2002 [108] Meijer et al. 2005 [80] Swaim et al. 2006 [97] Hortle et al. 2019 [54] Niu et al. 2019 [109] Ogryzko et al. 2019 [98]
	Infection biology	Adult Larvae Adult Larvae Larvae Adult, larvae	Prouty et al. 2003 [110] Cosma et al. 2006 [111] Harriff et al. 2007 [102] Takaki et al. 2013 [103] Johansen et al. 2018 [112] Johansen et al. 2018 [113]
*V. anguillarum*	Prophylactic approaches	Adult Adult Adult Adult Adult Larvae Larvae Larvae	Zhang et al. 2012 [40] Zhang et al. 2013 [41] Liu et al. 2014 [42] Zhang et al. 2014 [43] Gao et al. 2014 [44] Silva et al. 2014 [115] Caruffo et al. 2015 [116] Zhang et al. 2017 [117]
	Immunology	Adult Adult Adult Adult Adult Larvae	Zhang et al. 2012 [40] Zhang et al. 2013 [41] Liu et al. 2014 [42] Zhang et al. 2014 [43] Gao et al. 2014 [44] Oyarbide et al. 2015 [81]
	Infection biology	Larvae Larvae Adult Adult	O’Toole et al. 2004 [101] Oyarbide et al. 2015 [81] Liu et al. 2015 [118] Schmidt et al. 2017 [104]
*A. salmonicida*	Immunology	Adult Adult Adult, larvae	Lin et al. 2007 [82] Lin et al. 2009 [83] Da’as et al. 2011 [121]
*Y. ruckeri*	Prophylactic approaches	Adult, larvae	Korbut et al. 2016 [45]
	Immunology	Larvae	Sieger et al. 2009 [84]
	Infection biology	Larvae	Jank et al. 2015 [123]
*F. psychrophilum*	Prophylactic approaches	Larvae	Solís et al. 2015 [46]
	Immunology	Larvae	Solís et al. 2015 [46]

**Table 3:** Studies on viruses relevant for aquaculture utilizing zebrafish as an in vivo model.

Pathogen	Focus Area	Zebrafish Stage	Reference
SVCV	Prophylactic approaches	Adult Adult	Encinas et al. 2013 [55] García-Valtanen et al. 2014 [47]
	Immunology	Adult Adult, larvae Adult, larvae Larvae Adult Larvae Adult Larvae Adult Adult, larvae Adult, larvae Larve Adult Adult Adult	Levraud et al. 2007 [75] Aggad et al. 2009 [76] Aggad et al. 2010 [77] López-Muños et al. 2010 [85] Encinas et al. 2013 [55] Varela et al. 2014a [99] Varela et al. 2014b [86] Candel et al. 2015 [87] Li et al. 2015 [88] Pereiro et al. 2015 [89] Varela et al. 2016 [90] Espín-Palazón et al. 2016 [91] Feng et al. 2016 [92] Liu et al. 2019 [93] Medina-Gali et al. 2019 [138]
	Infection biology	Adult Adult	Sanders et al. 2003 [106] Wang et al. 2017 [131]
IHNV	Immunology	Adult, larvae Adult, larvae Larvae	Aggad et al. 2009 [76] Aggad et al. 2010 [77] Briolat et al. 2014 [48]
	Infection biology	Adult Larvae Larvae	LaPatra et al. 2000 [132] Liu et al. 2002 [133] Ludwig et al. 2011 [105]
VHSV	Prophylactic approaches	Adult Adult Adult	Novoa et al. 2006 [49] Chinchilla et al. 2013 [53] Kavaliauskis et al. 2015 [50]
	Immunology	Adult Adult, Larvae Adult	Encinas et al. 2010 [94] Dios et al. 2010 [95] Estepa and Coll 2015 [96]
	Infection biology	Adult	Kim et al. 2015 [136]
IPNV	Infection biology	Adult Adult	Seeley et al. 1977 [137] LaPatra et al. 2000 [132]

**Table 4:** Studies on parasite pathogens relevant for aquaculture utilizing zebrafish as an in vivo model.

Pathogen	Focus Area	Zebrafish Stage	Reference
*I. multifiliis*	*Immunology*	Adult Adult Adult Adult	Jørgensen 2016 [51] Christoffersen et al. 2016 [142] Jørgensen 2016 [100] Jørgensen et al. 2018 [52]
	*Infection biology*	Adult Adult	Cherry 2003 [143] Jørgensen 2016 [51]
*T. carassii*	*Infection biology*	Larvae	Dóró et al. 2019 [144]
